# Dissemination of a Novel Framework to Improve Blood Culture Use in Pediatric Critical Care

**DOI:** 10.1097/pq9.0000000000000112

**Published:** 2018-10-16

**Authors:** Charlotte Z. Woods-Hill, Laura Lee, Anping Xie, Anne F. King, Annie Voskertchian, Sybil A. Klaus, Michelle M. Smith, Marlene R. Miller, Elizabeth A. Colantuoni, James C. Fackler, Aaron M. Milstone

**Affiliations:** From the * The Children’s Hospital of Philadelphia, Philadelphia, Pa.; † The Leonard Davis Institute of Health Economics, University of Pennsylvania, Philadelphia, Pa.; ‡ Division of Pediatric Critical Care, Department of Pediatrics, University of Virginia School of Medicine, Charlottesville, Va.; § Department of Anesthesiology and Critical Care Medicine, Johns Hopkins University School of Medicine, Baltimore, Md.; ¶ Armstrong Institute for Patient Safety and Quality, Johns Hopkins University School of Medicine, Baltimore, Md.; ‖ Division of Infectious Diseases, Department of Pediatrics, Johns Hopkins University School of Medicine, Baltimore, Md.; ** MITRE Corporation, Mclean, Va.; †† Department of Pediatrics, Johns Hopkins University School of Medicine, Baltimore, Md.; ‡‡ The Johns Hopkins All Children’s Hospital, St Petersburg, Fla.; §§ Division of Quality and Safety, Department of Pediatrics, Johns Hopkins University School of Medicine, Baltimore, Md.; ¶¶ Department of Health Policy and Management, Johns Hopkins Bloomberg School of Public Health, Baltimore, Md.; ‖‖ Department of Biostatistics, Johns Hopkins Bloomberg School of Public Health, Baltimore, Md.

## Abstract

**Introduction:**

Single center work demonstrated a safe reduction in unnecessary blood culture use in critically ill children. Our objective was to develop and implement a customizable quality improvement framework to reduce unnecessary blood culture testing in critically ill children across diverse clinical settings and various institutions.

**Methods:**

Three pediatric intensive care units (14 bed medical/cardiac; 28 bed medical; 22 bed cardiac) in 2 institutions adapted and implemented a 5-part Blood Culture Improvement Framework, supported by a coordinating multidisciplinary team. Blood culture rates were compared for 24 months preimplementation to 24 months postimplementation.

**Results:**

Blood culture rates decreased from 13.3, 13.5, and 11.5 cultures per 100 patient-days preimplementation to 6.4, 9.1, and 8.3 cultures per 100 patient-days postimplementation for Unit A, B, and C, respectively; a decrease of 32% (95% confidence interval, 25–43%; *P* < 0.001) for the 3 units combined. Postimplementation, the proportion of total blood cultures drawn from central venous catheters decreased by 51% for the 3 units combined (95% confidence interval, 29–66%; *P* < 0.001). Notable difference between units included the identity and involvement of the project champion, adaptions of the clinical tools, and staff monitoring and communication of project progress. Qualitative data also revealed a core set of barriers and facilitators to behavior change around pediatric intensive care unit blood culture practices.

**Conclusions:**

Three pediatric intensive units adapted a novel 5-part improvement framework and successfully reduced blood culture use in critically ill children, demonstrating that different providers and practice environments can adapt diagnostic stewardship programs.

## Abstract 2

Supplemental Digital Content is available in the text.

## INTRODUCTION

Pediatric sepsis is a significant problem. A 2015 study across 128 pediatric sites in 26 countries reported an 8.2% prevalence of severe sepsis with an in-hospital mortality rate of 25%.^1^ Pediatric severe sepsis costs $4–5 billion per year, representing 16% annually of U.S. healthcare costs for pediatric hospitalizations.^2^ Early recognition and initiation of appropriate antibiotic therapy have been shown to impact sepsis-related outcomes significantly.^3^ Delayed initiation of appropriate antibiotics for patients with sepsis can lead to increased morbidity and mortality. Accordingly, the newest task force guidelines on sepsis ask clinicians to place urgent attention on rapid recognition and diagnosis of sepsis, but diagnostic tools for sepsis are imperfect.^4^

Clinicians regard blood cultures as a key test when considering the diagnosis of sepsis or bloodstream infection (BSI). Blood cultures, however, have a yield of only 5–15%, and up to half are falsely positive.^5–7^ These false-positive blood cultures represent diagnostic errors, lead to unnecessary antibiotics and additional testing, and are associated with increased lengths of stay and hospital costs.^8,9^ Importantly, increased antibiotic use may lead to antibiotic resistance.^10^ In addition, cultures from central venous catheters are more likely to be contaminated than those drawn from peripheral venipuncture, and failing to obtain cultures from peripheral venipuncture may both miss true BSI and overestimate the incidence of catheter-related BSI.^11–13^ Blood cultures, therefore, signify a prime opportunity to engage in diagnostic stewardship, an increasingly important construct in which refined used of diagnostic tools can decrease the utilization of tests, minimize false-positive results, and ultimately improve treatment decisions.^14^

Prior work at the Johns Hopkins Children’s Center (JHCC) has successfully and safely reduced blood culture use in critically ill children using clinical decision support tools.^15^ These tools consisted of a Fever/Sepsis Screening Checklist and a Blood Culture Decision Algorithm that clinicians would review and complete when considering ordering a blood culture. The JHCC project demonstrated a nearly 50% reduction in blood culture rate without increase in incidence of mortality, suspected sepsis, or suspected septic shock; and no increase in episodes of sepsis/septic shock in which patients received antibiotics in the absence of blood cultures.^15^ Based on this success, we subsequently implemented a multicenter quality improvement program to reduce unnecessary blood culture use in additional pediatric intensive care units (PICUs).

The objective of this project was to develop a customizable framework for implementation of a clinical approach to blood cultures intended to reduce unnecessary testing and to evaluate if this framework could successfully reduce blood culture use in critically ill children across diverse clinical settings and various institutions.

## METHODS

### Setting

Johns Hopkins University was the coordinating center, facilitating quality improvement initiatives at 3 ICUs in 2 study sites. The Johns Hopkins All Children’s Hospital is a tertiary care referral center in St. Petersburg, Florida, with a combined medical/surgical PICU and a separate cardiac ICU. The University of Virginia Children’s Hospital has a combined medical/surgical/cardiac ICU and is a tertiary care referral center in Charlottesville, Virginia. Each institution’s institutional review board acknowledged this quality improvement project and separately approved sharing of de-identified summary level data as nonhuman subjects research.

### Intervention

The intervention consisted of integrating the content of the clinical practice guideline that had been piloted, revised, and integrated into practice at the JHCC (the Fever/Sepsis Screening Checklist and the Blood Culture Decision Algorithm) into a novel 5-part Blood Culture Improvement Implementation Framework.^15^

This framework was built by a multidisciplinary team from JHCC with expertise in pediatric critical care, infectious disease, and human factors engineering and was intended to guide each participating unit through project adaptation and implementation. The general framework was identical for each unit, but local improvement teams tailored the operationalization of the individual steps to the specific environment. The 5 major components of the Blood Culture Improvement Implementation Framework included:

1) Completion of a work system assessment during an initial site visit. The Systems Engineering Initiative for Patient Safety (SEIPS 2.0) model guided creation of semistructured interviews. Qualitative content analysis of the interviews provided project teams with an understanding of current blood culture practices and helped identify potential barriers to adopting a new approach to blood cultures. The key findings of how people, tasks, physical environment, organization, and the external environment influence blood culture ordering decisions were then used to inform the rest of the implementation strategy.^16^ The interview guide is included as **Supplemental Digital Content C**, available at available at http://links.lww.com/PQ9/A48.

2) Identification of multidisciplinary stakeholders and a project champion. Each unit assembled a unique group of providers for leading the improvement project but integrated the expertise and support of faculty from disciplines such as nursing, patient safety/quality, critical care, infectious disease, cardiology, oncology, and surgery into the project team.

3) Collection and sharing of blood culture data. For internal benchmarking, each site first reviewed baseline blood culture rate data. Subsequently, sites established a system for routine transfer of electronic data to the coordinating center, to enable the JHCC team to monitor progress and to provide coaching and support.

4) Adaptation of clinician support tools by each unit. Each site received the 2-part JHCC clinical practice guideline in an editable format. Each unit revised and adapted the tools for the specific needs of their work environment and patient population. Although each team created different specific tools, there was a shared emphasis on raising awareness about potential negative consequences of unnecessary blood cultures, performing active consideration of risk factors for bacteremia, and evaluation for noninfectious causes of fever before blood culture decision.

5) Communication and analysis of progress throughout the implementation process, at varying frequencies, both within each site and between the sites and the coordinating center. Regular internal communication between the local site champion and the clinical teams throughout the project was strongly recommended by the coordinating center to facilitate successful implementation. Sites were encouraged to send weekly e-mails to attending physicians with feedback on the number of cultures sent on their patients as an important driver of reducing blood culture frequency, which had stood out as particularly effective in the JHCC implementation.^15^ In recognition of the different institutional cultures and workflow, the coordinating center did not dictate the exact type and frequency of internal communication about project progress.

The JHCC team performed initial site visits over 1–2 days to support the integration of this framework. The site visits included the formal work system assessment, review of the clinical practice guideline from JHCC and JHCC results, and question-answer sessions. Follow-up site visits occurred 10–12 months later. During the visits, participants reviewed project outcomes, challenges, and successes; shared particularly useful or high-impact strategies and discussed new or unanticipated barriers encountered during the implementation process.

### Outcome Measures

The primary quantitative outcomes were the number of blood cultures drawn per 100 patient days in each of the 3 units and the number of cultures drawn from central venous catheters (CVCs) versus peripheral venipuncture. Qualitative outcomes were 2-fold: the challenges and lessons learned during the implementation process from each unit; and how certain components of the implementation framework varied uniquely across the 3 units. Including these qualitative outcomes allowed the project team to understand drivers of successful versus unsuccessful behavior change around blood culture practices in the PICU setting.

### Statistical Analysis

To measure the effect of this quality improvement program on blood culture utilization, we compared patients admitted to each unit in the preintervention period of January 2014 to December 2015 to the postintervention period of January 2016 to December 2017. Statistical process control methods were used to evaluate changes in the blood culture rate over time. The initial centerline occurred at the arithmetic mean of the preintervention measurements. After setting control limits at 3 SDs from the mean rate, standard rules determined centerline changes.^17^

The effect of the program on blood culture utilization was additionally quantified by comparing blood culture rates per 100 patient-days in the pre- and postintervention periods based on a Poisson regression model for the number of blood cultures per month, which included the main term for intervention and an offset for the monthly patient-days. The Poisson regression model was fit using generalized estimating equations with an autoregressive within unit correlation structure and robust variance estimate to account for any misspecification of the within unit correlation structure and violation to the Poisson assumption (ie, overdispersion).

Finally, we applied a quasi-experimental interrupted-time series model to estimate (1) the relative change in the blood culture rates per month during the preintervention period; (2) the “immediate” effect of the intervention, reported as the relative change in the blood culture rate comparing the first month of the postintervention period with the last month of the preintervention period; and (3) the sustained effect of the intervention, reported as the relative change in the blood culture rate per month during the postintervention period. The interrupted-time series model estimates derive from extending the Poisson regression model described above to include the main term for time (linear), the main term for intervention, and the interaction between these 2 terms. The CVC blood culture rate was analyzed using the same methods as described above. Data were analyzed using Stata/IC (ver 15.0; StataCorp), and figures were generated using R (ver 3.4.1) and Excel.

## RESULTS

Table 1 describes clinical characteristics, census, and staffing models of each of the 3 units.

**Table 1.**
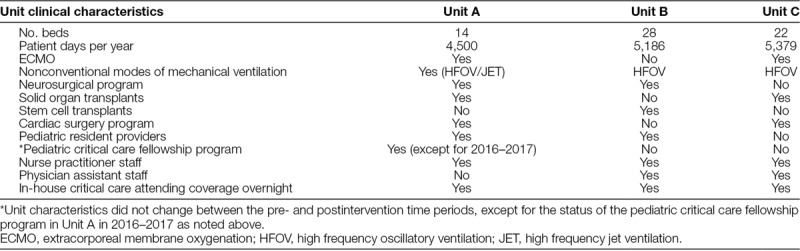
Clinical Characteristics of Participating Units

### Quantitative Outcomes

**Fig. 1.**
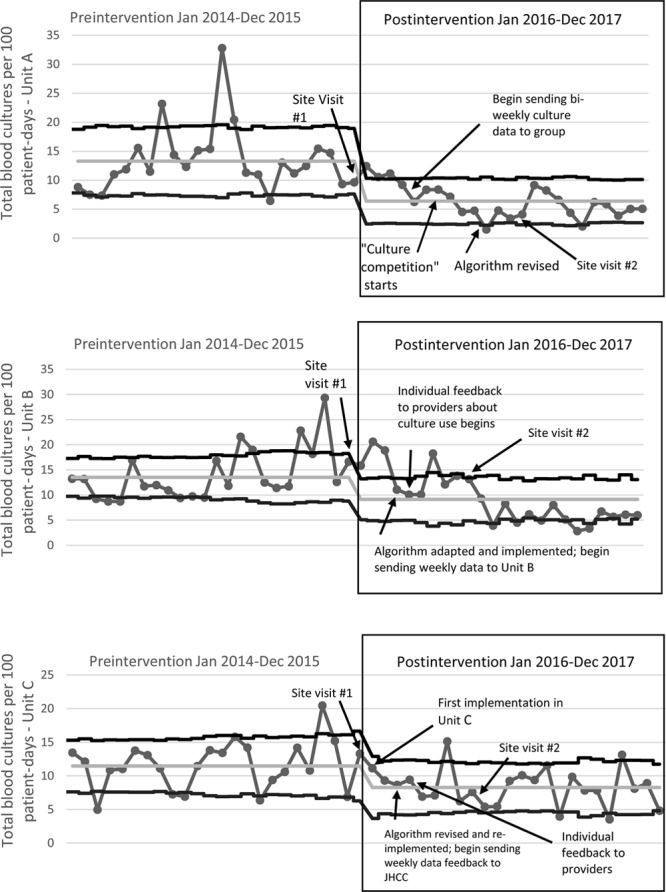
Rate of blood cultures in units A, B, and C before vs. after implementation of a quality improvement initiative to optimize use of blood cultures. Postintervention time period began January 2016.

During the preimplementation period, blood culture rates were 13.3, 13.5, and 11.5 per 100 patient-days, for Unit A, B, and C, respectively (Table 2, Fig. 1). Blood culture rates decreased to 6.4, 9.1, and 8.3 per 100 patient-days during the postimplementation period for Unit A, B, and C, respectively (Table 2, Fig. 1). Comparing postimplementation to preimplementation periods among all 3 units, the blood culture rate decreased by 32% [95% confidence interval (CI), 25–43%; *P* < 0.001].

**Table 2.**
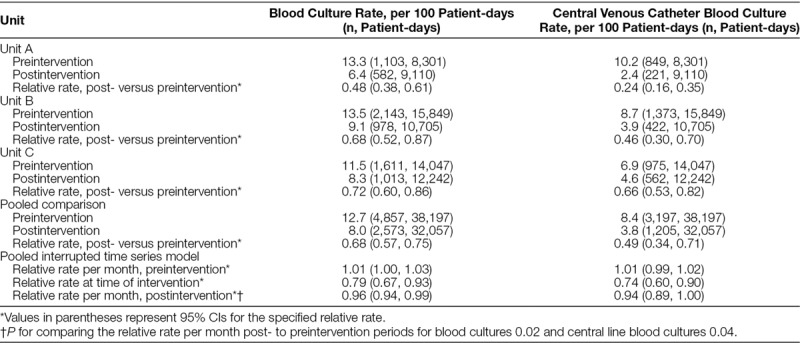
Total and Central Venous Catheter Blood Culture Rates per 100 Patient Days before versus after a Quality Improvement Initiative to Optimize Blood Culture Use in Units A, B, and C

Both Units A and B demonstrate a sustained reduction in blood culture rates, with postintervention average culture rates that remain within the new, decreased upper and lower control limits on Figure 1. Unit C, however, has more fluctuation and variability in their postintervention blood culture rates, indicating a lack of stable process control in this period (Fig. 1). The proportion of total blood cultures drawn from CVCs pre- versus postimplementation decreased from 77% to 38% (*P* < 0.001) in Unit A; from 64% to 43% (*P* < 0.001) in Unit B; and from 61% to 55% (*P* = 0.10) in Unit C). For all 3 units combined, there was a 51% reduction in central line blood culture rate in the postimplementation period (95% CI, 29–66%; *P* < 0.001; Table 2).

Interrupted time series analysis for the combined data for all units demonstrated a statistically significant decrease in the monthly change in the rate of total blood cultures during the postintervention period of 4% per month (95% CI, 1–6% decrease; *P* = 0.02; Table 2, **Supplemental Figure A**, available at http://links.lww.com/PQ9/A45). Analysis of the combined data also revealed a 6% per month decrease in central venous catheter blood culture rates (95% CI, 0–11% decrease; *P* = 0.04; Table 2, **Supplemental Figure B**, available at http://links.lww.com/PQ9/A46).

### Qualitative Outcomes

The interviews, focus groups, and communication records between the JHCC team and the study sites provided important qualitative data about the implementation process. Study teams across all sites encountered challenges during the implementation process. Table 3 summarizes the 4 primary challenges along with the corresponding solutions/lessons learned during the project.

**Table 3.**
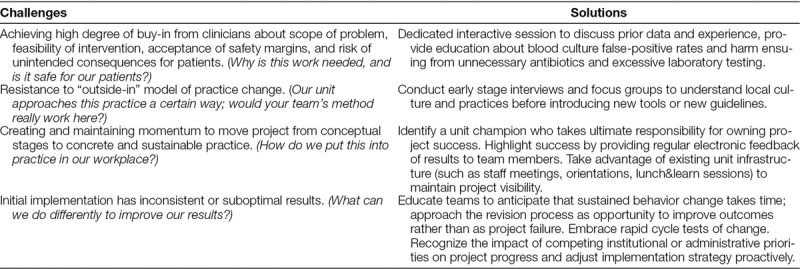
Qualitative Insights: Common Challenges and Potential Solutions for Blood Culture Optimization in Diverse Clinical Settings

Table 4 summarizes the unit-specific customizations for each component of this 5-part framework. Units were similar with regard to engagement in site visits, participation in the work system assessment, and data collection. The 3 units differed in the identity and involvement of the project champion, the adaptions of the clinical tools, and how project progress was monitored and communicated. Site-specific differences in implementation approach may have driven some of the observed variation in outcomes.

**Table 4.**
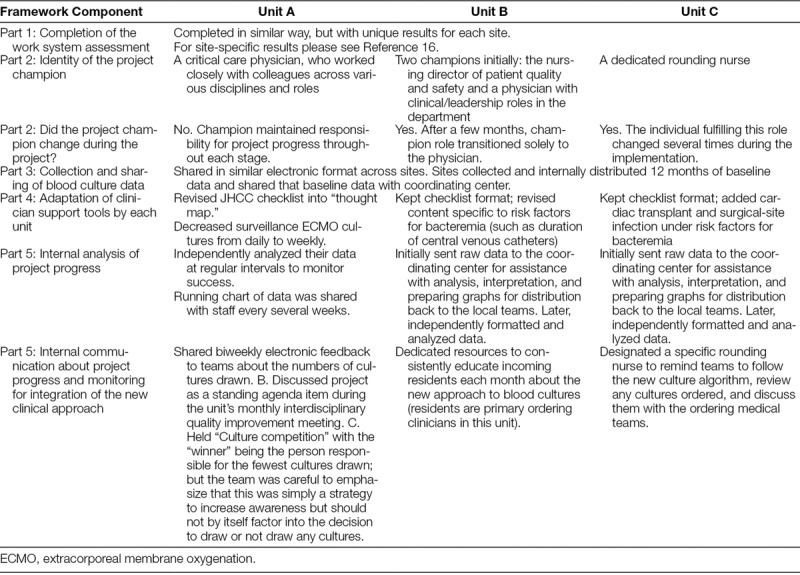
Unit-specific Approaches to the Customizable Implementation Framework

For example, in Unit A, a single local champion maintained continuous ownership of the project. The Unit A team monitored progress via independent internal data distribution and analysis. They maintained unit-wide awareness of the initiative by discussing the project during dedicated time at monthly meetings, and devised a “culture competition” as a unique implementation strategy to increase enthusiasm among clinicians. Unit A demonstrated sustained practice change without engaging a specific infectious disease champion and did not have any notable administrative barriers to participating in this project, such as changes in staffing or new leadership.

Unit B, in contrast, had more fragmented project ownership at first; though ultimately, consistent project champions emerged. Unit B also initially relied more than Unit A on the coordinating center for data feedback and analysis. Unit B faced the unique challenge of both a new staffing model and new unit leadership concurrent to project initiation. A high influx of newer staff members with greatly varying approaches to blood cultures made it challenging to engage, inform, and achieve the consistent and standardized practice that sites aimed to create. Like Unit A, the Unit B team did not have an infectious disease stakeholder take a leadership role in this work, though infectious disease faculty did participate in Unit B’s site visits. Unit B reported that not having more participation from this particular discipline made it challenging to achieve strong team buy-in, which was not the case for Unit A.

Finally, Unit C also experienced early challenges with maintaining consistent project champion engagement. The coordinating center performed much of the data organization and analysis for team C at first, with the site later independently managing its blood culture data. Like B, Unit C perceived its lack of infectious disease stakeholder involvement as a barrier to achieving team buy-in, and also faced some competing administrative priorities. For Unit C, this specifically took the form of separate high-profile quality improvement work focused on reducing contamination of blood culture specimens (part of existing central-line associated BSI reduction efforts). Effort devoted to this other work, along with changes to departmental leadership, made achieving the required focus on a separate, new culture reduction project more difficult.

## DISCUSSION

This project demonstrates that blood culture use can be reduced in critically ill children in a variety of clinical settings (medical/surgical PICU, cardiovascular intensive care unit [CVICU], and mixed medical/cardiac ICU) and across multiple institutions. JHCC project leads guided local teams using a novel 5-part framework and achieved significant practice change through site-specific revisions, flexibility in approach, and multidisciplinary collaboration. Including a formal work system assessment to inform implementation strategies was a unique strength of this project, demonstrating the critical role of human factors engineering principles in diagnostic stewardship initiatives.

There were important outcome differences between the units. Overall, the most robust results were in Unit A, compared with Unit C, which had a smaller decrease in blood culture rates and more variability in the postimplementation period. In the context of these quantitative results, the qualitative differences in implementation strategies across each site (Table 4) suggest important lessons for larger-scale implementation efforts. Certain factors or ingredients may be necessary for optimal outcomes of blood culture stewardship efforts, even as many components of the 5-part framework can be uniquely customized. A consistent project champion, strong unit-wide engagement, and awareness of competing organizational or administrative factors appears critical to successful, sustained practice change. In contrast, the role of infectious disease leadership is likely quite important, but may not be uniformly necessary for all institutions or all teams interested in launching this type of initiative. **Supplemental Digital Content D** (available at available at http://links.lww.com/PQ9/A47) summarizes these key factors into a brief primer on using the Blood Culture Improvement Implementation Framework. This may guide readers interested in beginning this type of improvement work in their own units.

Finally, the qualitative information summarized in Table 3 suggests a core set of issues that are universal to attempted behavior change around PICU blood culture practices: achieving an appropriate degree of multidisciplinary buy-in, accepting the project’s safety and feasibility, and tailoring the approach to the local work environment. Thematically, these emerged as fundamental tasks that each site needed to accomplish as part of the implementation process. How well these were mastered likely influenced the magnitude of project success. These preliminary insights will inform future larger scale implementation work by helping the study team to anticipate common concerns that local teams at prospective sites may raise and to prepare strategies to address these concerns proactively.

Participation of only 3 units limits the generalizability of our findings, and work to implement this project in a larger number of centers is currently underway. Analysis of only 24 months of postimplementation data may limit conclusions about sustainability, so ongoing monitoring is needed. The authors also acknowledge the importance of balancing metrics in quality improvement work. Previous work at JHCC answered important safety questions about reducing blood culture use in critically ill children.^15^ This project, in distinction, was focused on the development and dissemination of a quality improvement framework in diverse centers rather than re-demonstrating the outcomes or safety of the clinical approach. Participating units did not have the resources to repeat such an in-depth analysis of data about the incidence of sepsis or timely diagnosis of sepsis.

## CONCLUSIONS

Integrating a clinical practice guideline focused on diagnostic stewardship into a novel 5-part blood culture improvement framework facilitated site-specific, locally driven innovations and adaptations that successfully reduced blood culture use in 3 PICUs across 2 institutions. This project demonstrates that diagnostic stewardship is possible on a large scale in critically ill children, and takes important steps forward in reducing unintended patient harm from unnecessary testing. The potential downstream impacts of such a practice change, specifically regarding potential for reduced unnecessary hospital costs and reduced use of broad-spectrum antibiotics, are far-reaching and deserve further investigation.

## ACKNOWLEDGMENTS

The authors acknowledge Denise Remus from All Children’s Hospital for her valuable assistance in project implementation and staff engagement. The authors acknowledge the ICU staff in each participating unit for their commitment to quality improvement and patient safety.

## DISCLOSURE

The authors have no financial interest to declare in relation to the content of this article.

## Supplementary Material

SUPPLEMENTARY MATERIAL
