# Supplementary figures and images for: Dissemination of a Novel Framework to Improve Blood Culture Use in Pediatric Critical Care
Source: Pediatr Qual Saf. 2018 Oct 16;3(5):e112. doi: 10.1097/pq9.0000000000000112 (PMC6221585; doi:10.1097/pq9.0000000000000112)

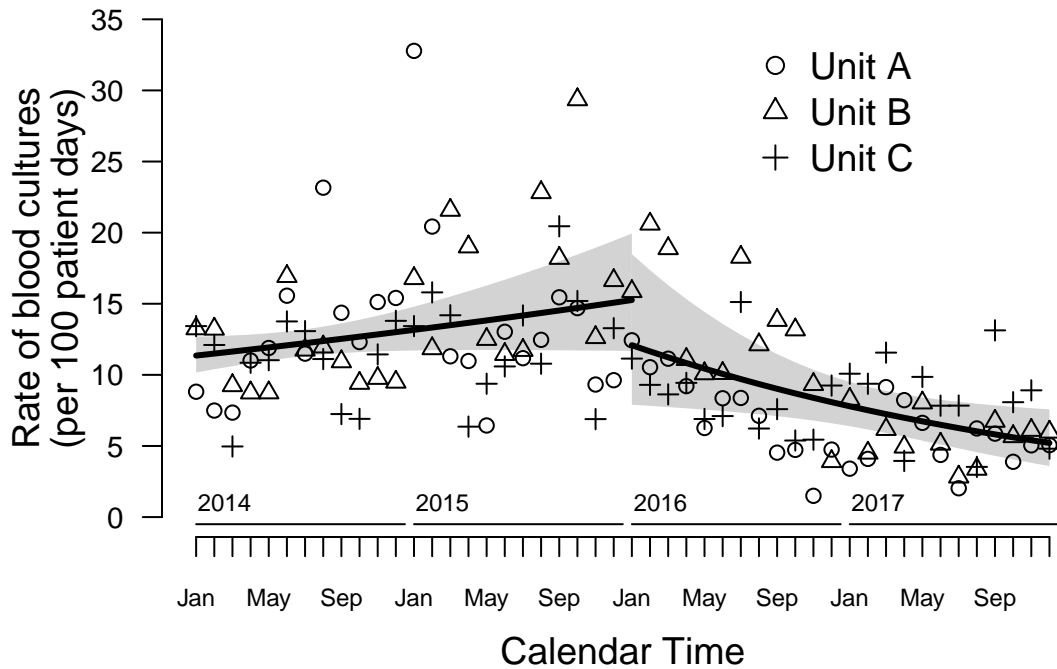

Supplement: SUPPLEMENTARY MATERIAL [file pqs-3-e112-s001.pdf]

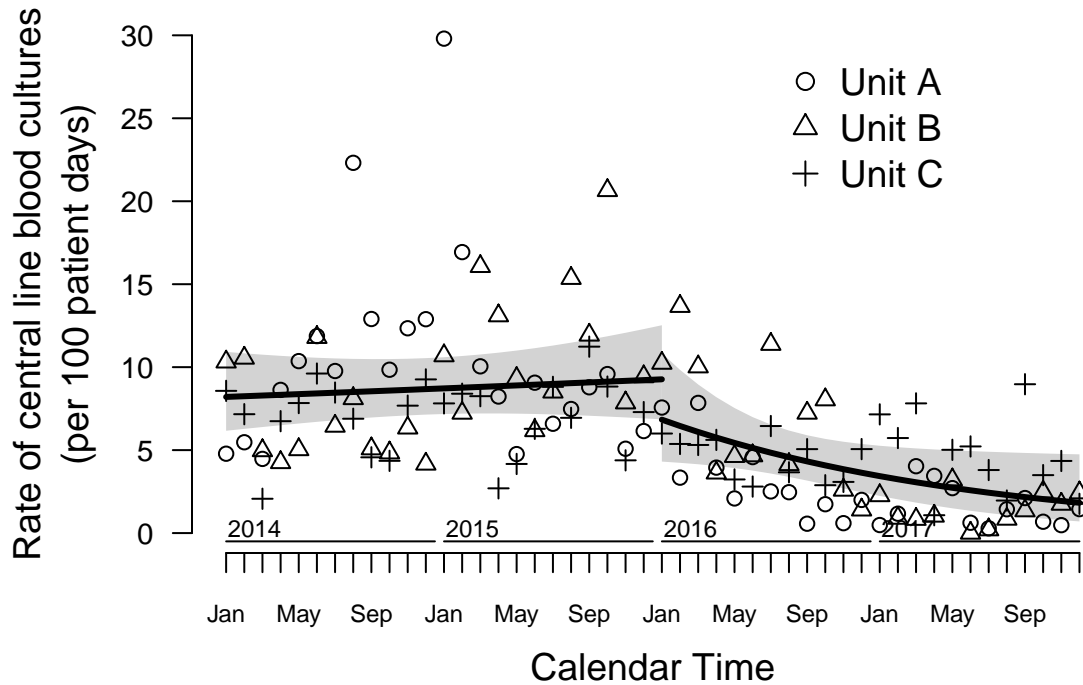

Supplement: SUPPLEMENTARY MATERIAL [file pqs-3-e112-s002.pdf]
