# Supplementary material for: Dissemination of a Novel Framework to Improve Blood Culture Use in Pediatric Critical Care
Source: Pediatr Qual Saf. 2018 Oct 16;3(5):e112. doi: 10.1097/pq9.0000000000000112 (PMC6221585; doi:10.1097/pq9.0000000000000112)
Supplement: SUPPLEMENTARY MATERIAL [file pqs-3-e112-s003.docx]

Supplement D: A brief primer on using the Blood Culture Improvement Implementation Framework

1. Required personnel: A dedicated project champion and a core project team

Project champion: A clinician familiar with the ICU environment who can devote time to the project in the pre-implementation and post-implementation periods. This clinician need not necessarily be a physician, but should partner with critical care and infectious disease physicians to ensure the new approach is grounded in the right clinical context for that ICU.

Core project team: 3-4 people with a combination of critical care, infectious disease, nursing, and safety/quality expertise; though this can vary.

1. Required data elements: blood culture rate (number of blood cultures ordered and number of patient days in the ICU)
2. Time commitment for the project champion and core project team: This will vary site to site, but much of the time involved will be spent in the pre-implementation phase on the tasks listed below, particularly for discussions with stakeholders and creation of the clinical tools. Approximately 1-2 hours every 1-2 weeks is a general estimate. After implementation, depending on the initial results and methods used for auditing cultures, about 1 hour every 1-2 weeks may be sufficient, with less time needed as the approach becomes more integrated into usual clinical practice.
3. Pre-implementation tasks:
4. Examining the unit’s baseline blood culture rate data before implementation, and establishing a system for how to analyze that data in each phase of the project. This need not be more complex than looking at number of blood cultures/number of patient days for all included ICU patients, and we suggest analyzing this on a weekly basis.
5. Understanding the current/baseline approach to blood cultures in the unit (please see the supplement Interview Guide and Dr. Xie’s paper “Work system assessment to facilitate the dissemination of a quality improvement program for optimizing blood culture use: a case study using a human factors engineering approach” cited in the references for one example of how to accomplish this).
6. Reaching out to key stakeholders whose perspectives should inform your new approach (such as Oncology or Transplant teams).
7. Coming to consensus on the clinician support tools your unit will implement.
8. Developing strategies for educating your providers about the new approach to blood cultures and raising awareness about the project. Examples include targeted emails to ordering providers, short discussions at unit conferences or staff meetings, online learning modules, and posting descriptive flyers as well as the tools themselves (if using a paper format) around the workspaces in the unit. Embedding the clinical tools into the electronic medical record, if possible, is another possibility.
9. Post-implementation tasks:
10. Analyzing the blood culture rate using a run or control chart if possible; and doing so on a weekly basis to monitor progress.
11. Sharing this data with your unit’s clinicians, using feedback to help drive behavior change. This can be accomplished simply by emailing the weekly control charts to your clinicians, or by targeted emails to attending physicians displaying the number of blood cultures sent for their patients that week or that month.
12. Monitoring for adherence to the new guidelines and for unintended consequences. One example strategy is to audit blood cultures ordered in the unit for a brief period of time (such as 1-2 months after implementation starts) to see if clinicians are following the guidelines; and to audit positive blood cultures after implementation starts to see if there was any delay in appropriately obtaining a blood culture or treating infection.
13. Revising the clinical approach or implementation plan if initial results are suboptimal. A key part of quality improvement is the Plan-Do-Study-Act rapid test of change model. The project champion and core project team should be prepared to alter their approach and try new things to optimize this improvement work in their unit.
