# Supplementary material for: Dissemination of a Novel Framework to Improve Blood Culture Use in Pediatric Critical Care
Source: Pediatr Qual Saf. 2018 Oct 16;3(5):e112. doi: 10.1097/pq9.0000000000000112 (PMC6221585; doi:10.1097/pq9.0000000000000112)
Supplement: SUPPLEMENTARY MATERIAL [file pqs-3-e112-s004.docx]

Supplement C. Interview guide used during work system assessment of PICU blood culture practices.

Interview Guide

Total duration:

NOTE: All questions are open-ended to encourage the interviewee to discuss topics related to the study. In such discussions, additional questions and prompts may be used to encourage the interviewee to fully explain his or her answer. These questions and prompts include “Can you tell me a bit more?” “I’m not sure I quite understand about [repeat respondent’s words],” “You said [repeat respondent’s words], could I ask you a bit more about that?” or “Could you explain more about what you meant in saying [repeat respondent’s words].”

Introduction

Thank you for agreeing to participate in the interview. We are interested in understanding your experience with blood culture ordering in the pediatric setting.

Do you have any questions about the study? Are you willing to proceed with the interview? Do you mind if we audiotape the interview?

Reminder: Any examples (based on your previous experiences with blood culture ordering) you can provide that help me understand your responses to my questions would be helpful.

Blood culture practices:

- In general, how is a decision on whether or not to order a blood culture made?
  - What are the main reasons for obtaining a blood culture?
  - What information is needed to make the decision on whether or not to order a blood culture? Where and how do you get the information you need to make the decision?
  - Can you think of a situation when you ordered a blood culture that could have not been ordered?
  - Can you think of a situation when you did not but would have ordered a blood culture?
- From what source is a blood culture usually obtained, central line, arterial line, or peripheral venipuncture?
  - When do you think a blood culture should be drawn from the central line?
  - When do you think a blood culture should be drawn from the arterial line?
  - When do you think a blood culture should be drawn from the peripheral venipuncture?
- What is your role in decision-making on ordering a blood culture? Who else are involved in blood culture ordering decision-making?
  - How does the healthcare team collaborate to make decision on ordering a blood culture?
- What are the other work system factors that can influence your decision on whether or not to order a blood culture? What facilitate or hinder your decision on whether or not to order a blood culture?
  - What are the tools you use to support your decision-making on blood culture ordering?
  - How would the physical environment of a unit influence your decision-making on blood culture ordering?
  - Do you have a protocol for ordering blood culture in your institute? If yes, how do you use the protocol?

Fever Checklist Tool and Blood Culture Decision Algorithm:

Introducing the fever checklist tool and the blood culture decision algorithm to the participant and going through each of the tools with the participant

- What do you think about the checklist/algorithm? What makes sense to you? What does not make sense to you? Why?
  - What is it about the checklist/algorithm that is (not) useful? Why?
  - What is it about the checklist/algorithm that you like or dislike (e.g., content, format)? Why?
  - What information on the checklist/algorithm you think is (not) important to making decisions on blood culture? Why?
  - What are your suggestions to improve the checklist/algorithm?
- How would you use the checklist/algorithm to support your decision-making on blood culture?
  - How would the checklist/algorithm fit into your current work system and processes?
  - What would be the challenges to using the checklist/algorithm to support your decision-making on blood culture in your daily practices?
  - What would be the facilitators and barriers to using the checklist/algorithm?
  - What are the potential strategies for addressing each of the challenges?

Final questions:

- Is there any other information you would like to share regarding blood culture practices?
- What else do you think I should be asking you about?
